# Breakthrough in efficient cloning and activation of large cryptic biosynthetic gene clusters from high GC actinobacteria

**DOI:** 10.1016/j.synbio.2022.06.006

**Published:** 2022-06-22

**Authors:** Jun-Yi Wang, Bing-Zhi Li

**Affiliations:** Frontiers Science Center for Synthetic Biology and Key Laboratory of Systems Bioengineering (Ministry of Education), School of Chemical Engineering and Technology, Tianjin University, Tianjin, 300072, PR China

**Fig. 1:**
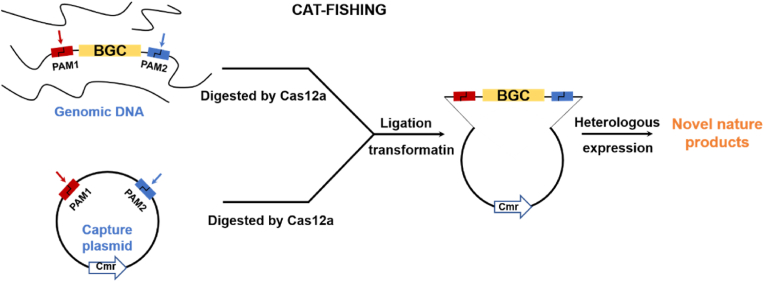
Schematic diagram of CAT-FISHING. BGC: biosynthetic gene cluster, PAM: protospacer adjacent motif.

Synthetic genomics, as one of the important parts of synthetic biology, has become a hotspot in recent years, and several bacterial genomes and yeast chromosomes have been designed and synthesized from *de novo*. However, during genome synthesis, one technical challenge is the efficient cloning and editing of large genome fragments. At the same time, with the advent of the genomics era, actinobacteria (e.g. *Streptomyces*) is again regarded as a rich source for novel natural products. However, most of Streptomyces and nearly half the actinomycetes are high GC content. And the capture, editing and activation of large gene clusters with high GC content is also a major difficulty in the functional mining of cryptic gene clusters. In a breakthrough article recently reported in *Nucleic Acids Research*, Liang et al. has successfully developed a method to efficiently clone and edit large fragments of DNA in actinomycete genomes, called CAT-FISHING (CRISPR/Cas12a-mediated fast direct biosynthetic gene cluster cloning) [1]. Combining the DNA-cleaving activity of Cas12a [2], the unique advantages of BAC library construction, and the high efficiency of *E. coli* DNA ligase, they developed a phenomenal method for the rapid and direct cloning of large biosynthetic gene clusters with high GC content (Fig. 1).

Bacterial artificial chromosome (BAC) library construction technology is indiscriminate in the DNA sequence of insertion, and thus suitable for cloning of large DNA fragment with high GC contents [3]. However, using the restriction enzymes to partially and randomly digest the genomic DNA in BAC library construction makes the cloning procedure is time consuming and labor intensive. In order to eliminate the effect of restriction endonuclease cleavage sites contained in gene fragments on cloning, the combination of CRISPR/Cas and traditional assembly techniques has spawned a series of new gene cluster cloning methods. For example, CATCH technology based on CRISPR/Cas9 and Gibson assembly [4], ExoCET technology formed by integrating CRISPR/Cas9 and RecET [5], CAPTURE technology using CRISPR/Cas12a and loxP site recombination [6], etc. In addition, TAPE technology based on TelN/tos and Gibson assembly is also a facile and efficient method for large fragment assembly [7].

In this work, CAT-FISHING uses CRISPR/Cas12a to replace the restriction enzymes that used to construct bacterial artificial chromosomes. Under the guidance of crRNA pairs, Cas12a not only precisely cuts out the target gene cluster from the high-quality actinomycete genomic DNA prepared by agarose gel embedding (Lysis and digestion directly in low melting point agarose gel blocks, largely avoiding mechanical shearing and yielding high quality genomic DNA samples), but also generated two sticky ends with 4- or 5-nt overhangs flanked the gene cluster fragment. Then, under the assistance of DNA ligase, the cohesive ends of gene cluster fragments could ligate to BAC plasmid, and realize the direct cloning in vitro. The application of Cas12a in this technology not only shortens the cloning steps, making the steps simpler and less time-consuming, but also reduces the cost of cloning, making it a powerful choice for batch cloning of gene clusters.

Among them, pulsed-field gel electrophoresis (PFGE) is an important and powerful tool for separating large DNA fragments, but this step is time-consuming and labor-intensive. To rapidly clone large BGCs directly from genomic DNA, this method developed a PFGE-free procedure that greatly reduced experimental time. After Cas12a digestion, the resulting mixture containing genomic DNA can be used directly for subsequent ligation and transformation. Using a PFGE-free procedure, they successfully cloned high GC content BGCs of different sizes, and the size of the fragment affected the efficiency of the cloning. Optionally, the isolation and purification of target DNA fragments by PFGE significantly improved the cloning efficiency of target BGCs, close to the cloning efficiency of the CAPTURE method [6].

CAT-FISHING was applied to directly clone targeted BGCs from different actinomycete genomic DNA samples, which indicated the universality of CAT-FISHING in actinomycetes. Actinomycetes are widely used to produce a variety of bioactive small molecules such as avermectin [8,9], proving their great potential in the production of new drugs. And there are a large number of silenced gene clusters in the actinomycete genome. Activation of these silenced gene clusters through heterologous expression may aid the search for natural product (NP)-based drug. Eight gene clusters of different sizes were captured from six different actinomycetes. It is worth mentioning that a 145-kb DNA fragment with a GC content of 75% was successfully captured. To our knowledge, this is the largest DNA fragment ever obtained by in vitro direct simple cloning from genomic DNA with such high GC content. In the future, CAT-FISHING can be used in the synthesis of genome with high GC content.

Furthermore, a 110kb cryptic gene cluster cloned from a *Micromonospora* strain using the CAT-FISHING was expressed heterologously in the *Streptomyces* chassis, and a new macrolide amine compound marinolactam A was obtained. The compound has moderate anticancer activity against HCT116 human colon cancer cells. This provides an inspiring example of this approach for cloning, editing and activating the large silent gene clusters in actinomycetes, leading to the discovery of active novel natural products. And it would be an important asset to the entire community of natural product-based drug discovery.
